# Embolism of the Central Artery of the Retina during the Injection of Paraffin

**Published:** 1903-08-01

**Authors:** 


### EMBOLISM OF THE CENTRAL ARTERY OF THE RETINA DURING THE INJECTION OF PARAFFIN.

That there is some slight danger of embolism following the injection of paraffin in order to remedy deformities is well recognised, though with due precautions, such as keeping the patient quiet in bed and using a paraffin which has not got a low melting point, the likelihood of such an accident occurring appears to be exceedingly small. A strange case has lately been recorded by Drs. L. M. Hurd and W. A. Holden,[1] of New York, of embolism of the central artery of the retina occurring while the operation was proceeding. The patient, a healthy man of 32 years of age, had twice previously had paraffin injected in order to remedy a deformity of his nose following the destruction of the cartilaginous septum. On the third occasion he requested that a little more paraffin might be introduced near the site of the second injection. A mixture of paraffin with a melting point of 130° Fahr. and ordinary white vaseline, having together a melting point of 110° Fahr., of a semi-solid consistency, was accordingly injected at the root of the nose. While this was going on the patient was seen to rub his right eye, and in reply to a question he said that he could not see with it. A little later ecchymosis appeared about the tip of the nose, showing that a vein had been punctured. Twenty-five minutes afterwards the eyes were examined. The pupil of the right eye was large and did not respond to light, and the patient had subjective sensations of objects swimming about in the entire field of vision, but had lost perception of light. The media were clear, the retina was not hazy, and the retinal veins appeared normal. The main inferior branch of the central artery of the retina and its divisions, however, were empty and collapsed, being recognisable only by the faint white outlines of their walls. The main superior branch contained some blood, but when gentle pressure was made upon the eyeball, the blood column here broke up and the blood flowed back into the central artery. Two hours later the optic disc had become slightly blurred at its margins and the retina about the larger vessels was hazy. The usual red spot at the macula was clearly marked. Both arteries and veins were empty of blood. This was probably due to the fact that massage had been applied just before. The case is interesting from two points of view; in the first place it afforded an excellent opportunity for observing the early changes following embolism of the central artery of the retina, and secondly there are such difficulties in the way of explaining satisfactorily how the embolus got there. It is not easy to suppose that a foreign body large enough to block the central artery could have first found its way through the capillaries of the lung. The possibility of there being a persistent foramen ovale between the two auricles was suggested as a possibility. Of course, it is also conceivable that the plugging was a coincidence, and that the embolus consisted of clot and not of paraffin. There is one other case recorded of blindness following the injection of paraffin in order to remedy the deformity due to a sunken nose, but in that case the lesion appeared to be thrombosis of the ophthalmic vein.

[1] Medical Record, July 11.

